# After the Hurricane: Anti-COVID-19 Drugs Development, Molecular Mechanisms of Action and Future Perspectives

**DOI:** 10.3390/ijms25020739

**Published:** 2024-01-06

**Authors:** Hazim O. Khalifa, Yousef M. Al Ramahi

**Affiliations:** 1 Department of Veterinary Medicine, College of Agriculture and Veterinary Medicine, United Arab Emirates University, Al Ain P.O. Box 1555, United Arab Emirates; 201809551@uaeu.ac.ae; 2 Department of Pharmacology, Faculty of Veterinary Medicine, Kafrelsheikh University, Kafrelsheikh 33516, Egypt

**Keywords:** SARS-CoV-2, COVID-19, anti-COVID-19, viral inhibitors, anti-COVID-19 mechanism of action, monoclonal antibodies

## Abstract

Severe Acute Respiratory Syndrome Coronavirus-2 (SARS-CoV-2) is a new coronavirus in the *Coronaviridae* family. The COVID-19 pandemic, caused by SARS-CoV-2, has undoubtedly been the largest crisis of the twenty-first century, resulting in over 6.8 million deaths and 686 million confirmed cases, creating a global public health issue. Hundreds of notable articles have been published since the onset of this pandemic to justify the cause of viral spread, viable preventive measures, and future therapeutic approaches. As a result, this review was developed to provide a summary of the current anti-COVID-19 drugs, as well as their timeline, molecular mode of action, and efficacy. It also sheds light on potential future treatment options. Several medications, notably hydroxychloroquine and lopinavir/ritonavir, were initially claimed to be effective in the treatment of SARS-CoV-2 but eventually demonstrated inadequate activity, and the Food and Drug Administration (FDA) withdrew hydroxychloroquine. Clinical trials and investigations, on the other hand, have demonstrated the efficacy of remdesivir, convalescent plasma, and monoclonal antibodies, 6-Thioguanine, hepatitis C protease inhibitors, and molnupiravir. Other therapeutics, including inhaled medicines, flavonoids, and aptamers, could pave the way for the creation of novel anti-COVID-19 therapies. As future pandemics are unavoidable, this article urges immediate action and extensive research efforts to develop potent specialized anti-COVID-19 medications.

## 1. Introduction

SARS-CoV-2, which is responsible for the COVID-19 outbreak, is not the first coronavirus to pose a significant public health risk. During the previous 20 years, the severe acute respiratory syndrome coronavirus (SARS-CoV) in 2002, H1N1 influenza in 2009, and the Middle East respiratory syndrome coronavirus (MERS-CoV) in 2012 have emerged [1]. The World Health Organization (WHO) was notified about an outbreak of the novel SARS-CoV-2 virus in Wuhan, China, in December 2019 [2]. It was initially termed “2019-nCoV”, but the International Committee on Taxonomy of Viruses renamed it SARS-CoV-2 on 11 February 2020 due to its similarities to SARS-CoV [3]. Because of the rapid and uncontrolled spread of the virus, the WHO proclaimed it a global pandemic on 11 March 2020 [4]. The mortality rate of SARS-CoV-2 is lower than that of SARS-CoV, with 6.76% and 9.6%, respectively [5]; meanwhile, the rate of transmission of SARS-CoV-2 is higher than that of SARS-CoV. The WHO has reported more than 772.8 million cases of SARS-CoV-2 infection worldwide as of 17 December 2023, including more than 6.9 million deaths “https://covid19.who.int/ (accessed on 22 December 2023)”. Furthermore, the outbreak was linked to massive economic losses all across the world. According to a recent estimate, a 20% COVID-19 infection rate in the United States based on a Monte Carlo simulation resulted in a total direct medical cost of $163.4 billion throughout the course of the pandemic [6]. The primary causes of higher costs were consistent across countries and included intensive care unit (ICU) admission and in-hospital resource utilization such as mechanical ventilation, which resulted in cost increases ranging from $2082.65 ± 345.04 to $2990.76 ± 545.98 [6]. Since it was declared a public health danger, the initial main hurdle has been creating diagnostic methods to correctly identify the existence of the virus and generating effective treatments to combat SARS-CoV-2. This however was not the case, as clinical trials and studies demonstrated most of these medications have failed to show any or limited efficacies. Fortunately, some were approved by the FDA due to their effectiveness and are a part of the treatment plan against SARS-CoV-2. Needless to say, the search for effective antivirals for SARS-CoV-2 continues to this day. Therefore, the present review was developed to discuss the development and progress of SARS-CoV-2 antivirals. Furthermore, we discussed in depth the molecular mechanism of action of generally approved anti-COVID-19 drugs in this review, as well as some potential therapeutic options that may involve a breakthrough in the development of anti-COVID-19 therapy.

## 2. The Origin of COVID-19

Coronaviruses have been known to exist for nearly a century. The first coronavirus discovered was avian infectious bronchitis virus (IBV) in 1937, followed by the discovery of murine hepatitis virus 10 years later [7]. In contrast, the first case of human coronavirus (HCoV-229E) was found in 1965 by virologists Tyrrell and Bynoe [8]. This was accomplished by collecting nose swabs from medical students who displayed symptoms of a typical cold with nasal discharge during a respiratory illness investigation in which one sample contained the virus [7]. The virus had morphological similarities to the IBV, which was discovered a while ago when inoculated in an organ culture. Their research concluded that common colds can be transmitted via nasal secretions. Coronaviruses have a reputation for rapidly evolving. As a result, they risk infecting many species and possibly causing zoonosis. SARS-CoV-2 is the ninth coronavirus shown to be capable of infecting humans. It is also the seventh coronavirus discovered in the last 20 years [9]. Despite this, coronaviruses have been identified as a high-risk infection capable of causing a pandemic due to their great transmission capabilities. All previous human coronaviruses were linked back to animal origins, giving an understanding of where SARS-CoV-2 originated. SARS-CoV-2 was first identified in Wuhan, central China. The first cases in Wuhan, China, are thought to have been caused by a zoonotic source, notably the Huanan market, which is known to sell dogs, bats, snakes, poultry, and fish [10]. Two of the first three COVID-19 cases were directly linked to the Huanan market. Furthermore, 28% of COVID-19 cases identified in December 2019 were traced back to the same market [9]. Despite the fact that the early cases were tied to the Huanan market, subsequent cases had ties to other markets, and some had no ties to any market at all. As a result, no definitive conclusion could be drawn about the Huanan market, whether it was an amplifier or the source of SARS-CoV-2. Extensive molecular and genetic testing was performed on viruses gathered from various animals, and it was discovered that coronaviruses very similar to SARS-CoV-2 were isolated in bats and pangolins [11]. This could imply that bats and pangolins are reservoir hosts for the virus that gave rise to SARS-CoV-2. It should be noted that none of the viruses obtained from bats or pangolins are sufficiently similar to SARS-CoV-2 and hence cannot be its source [11].

## 3. Viral Structure

The *Coronaviridae* family is divided into two subfamilies: the *Coronavirinae* and the *Torovirinae*. Coronaviruses are members of the order *Nidovirales* and the subfamily *Coronavirinae*. Coronaviruses are classified into four genera: alpha, beta, gamma, and delta. SARS-CoV-2, like SARS-CoV and MERS-CoV, is classified as a beta-coronavirus [12]. SARS-CoV-2’s genome is a single-stranded positive-sense RNA virus (~29.9 kb), which is larger than any previous RNA virus [12]. Transmission electron microscopy and scanning electron microscopy were used to determine the structural identity of SARS-CoV-2 [13]. The electron microscope scans revealed a crown-shaped structure, hence the name “Corona”. The viral particle measured 60–140 nm in size [13]. SARS-CoV-2 is made up of four structural proteins (S, M, E, N) and sixteen non-structural proteins (nsp1–16), each having its own particular function. Having said that, SARS-CoV-2, a beta-coronavirus, has three essential envelope proteins. The first envelope protein is the spike protein (S), which aids in attachment to cell membrane receptors, membrane fusion, and finally, entrance into the host cell. As a result, it is an appealing target for antivirals. S1 and S2 are the two components of the (S) protein. The S1 subunit’s function is to attach to the host cell’s receptor. Meanwhile, S2 is in charge of the viral and host cell fusion. The structure of the coronavirus membrane is determined by the second envelope protein, the membrane protein (M), and the third protein, the envelope protein (E). Another protein that is not an envelope protein but is nevertheless very important is the nucleocapsid protein (N), which is a structural protein that forms complexes with genomic RNA and contributes to virus transcription and assembly [14].

## 4. SARS-CoV-2 Replication

### 4.1. Attachment and Entry

It all starts when a person inhales an airborne viral particle (SARS-CoV-2). The viral particle will move through the airway, where it will interact with the epithelial cells. As previously stated, the spike protein of SARS-CoV-2 plays a key role in cell attachment, fusion, and, finally, entry into the host’s cell [15]. As a result, the host-pathogen interaction begins with spike protein binding to the angiotensin-converting enzyme-2 (ACE2) receptor (the cellular receptor of SARS-CoV-2). Although ACE2 receptors can be found on the surfaces of many organs, including the lung, endothelium, kidney (proximal convoluted tubules), heart, testis, bladder, and intestines, alveolar epithelial type II cells are the most prevalent (83%) [13,15]. Because alveolar epithelial type II cells contain the bulk of ACE2 receptor-presenting cells, this could explain SARS-CoV-2’s predilection for the lungs. After binding to the ACE2 receptor, the S protein becomes locked in, allowing another protein expressed on the cell’s surface to cleave the spike protein at certain places. This is achieved by the enzyme transmembrane serine protease 2 (TMPRSS2), which is required for the spike protein to be activated, resulting in viral membrane fusion or endosomal entry [13,15].

### 4.2. Replication

After fusional entrance or receptor-mediated endocytosis, the viral genome (RNA) is released into the host’s cytoplasm. Once the positive sense RNA penetrates the host cell’s cytoplasm, translation begins. It is translated into viral polyproteins by the ribosome of the host cell. The viral polyproteins are broken down by 3CLpro (3C-like protease) and PL^pro^ (papain-like protease) into non-structural proteins (nsp) that form the transcription complex [16]. The RNA-dependent RNA polymerase (RdRp) active site is held by nsp12, which requires the accessory components nsp7 and nsp8 to be enzymatically active [17]. During replication, RdRp, a large protein complex and a critical enzyme, catalyzes the creation of new viral RNA. Translation happens once again, producing S, M, E, and N proteins as well as a number of auxiliary proteins [18]. The N protein will bind to the genomic RNA, unlike the S, M, and E proteins. The three latter proteins will bind to the endoplasmic reticulum membrane (ER), forming the endoplasmic reticulum-Golgi intermediate compartment (ERGIC), also known as the vesicular-tubular cluster. Finally, the viral genome encased in its nucleocapsid is brought to the lumen of the ER and encapsulated, and the new virus (daughter virus) is transferred by the ERGIC to the host cell’s plasma membrane and discharged via exocytosis [19].

## 5. Anti-COVID-19 Drug Development

### 5.1. Solidarity Trial

The WHO classified SARS-CoV-2 as a Public Health Emergency of International Concern on 30 January 2020 [20]. This was ongoing, with thousands of new cases reported daily in Wuhan, China, and tens of thousands more reported from countries around the world, including Thailand, Japan, South Korea, the United States, France, Germany, the United Arab Emirates, India, Italy, Russia, the United Kingdom, Malaysia, and Canada. After about 6 weeks, the WHO launched the “Solidarity Trial”, a global clinical trial, in March [21]. It is an initiative to identify effective SARS-CoV-2 therapies. A new medicine typically takes 1–2 decades to produce due to safety standards and efficacy criteria. When dealing with a highly contagious disease or a pandemic, a better method would be to repurpose a previously available medicine on the market that shares similar mechanisms (same targets) as the virus. Remdesivir, lopinavir/ritonavir, lopinavir/ritonavir plus interferon beta, and chloroquine were the four most promising medicines at the commencement of the “Solidarity Trial” [21]. The trial aimed to test the efficacy of these four most promising treatments versus the global standard of care against SARS-CoV-2. To acquire the most precise numerical data, it was critical that the selected patients be from all age groups, nationalities, and health statuses. The goal was to determine which of the four medications could halt the advancement of the disease or, better yet, enhance the patient’s survivability rate. More medications can be added to the “Solidarity Trial” for testing based on developing fresh findings. The trial included countries from all around the world, with approximately 90 countries taking part.

### 5.2. Timeline of Drug Development

**Figure 1:**
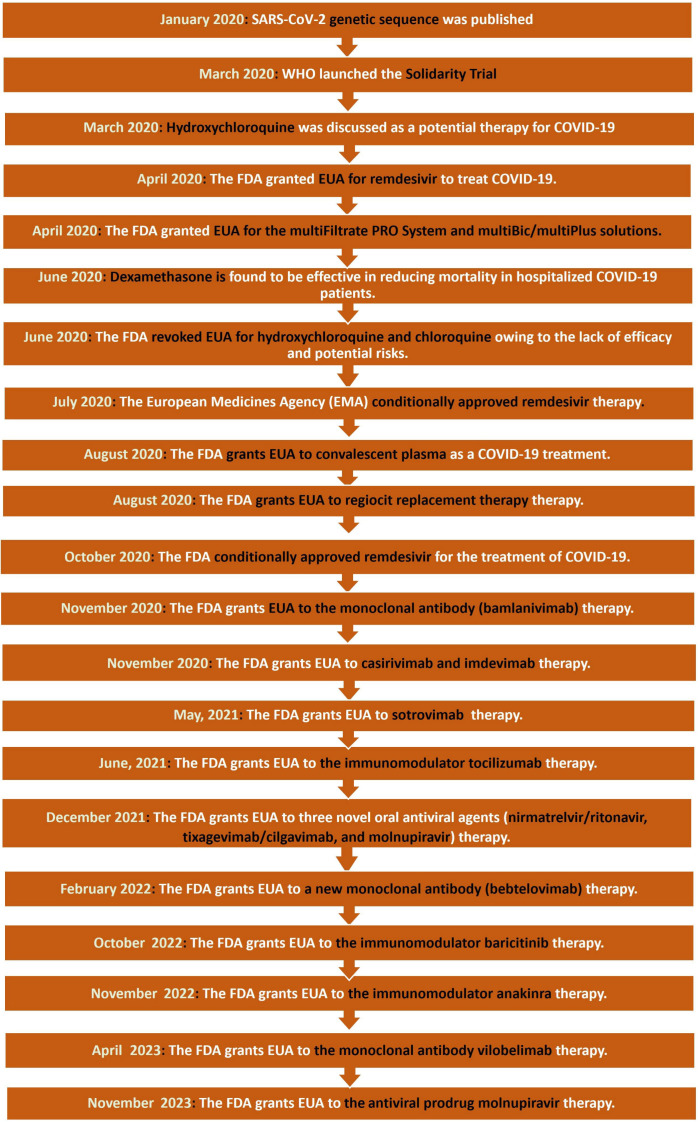
Timeline for the development of some important anti-COVID-19 approved therapy.

When it comes to combating a pandemic or disease, the first step is to study the pathogen’s genetic and structural properties. This is extremely important for generating an accurate diagnostic tool, researching the virus, and producing new treatments and vaccines. On 5 January 2020, a team of researchers from the Shanghai Public Health Clinical Center and Fudan University’s School of Public Health performed genome sequencing of SARS-CoV-2 (Figure 1) [22]. To accomplish this, a technique known as shotgun sequencing was used, which involves breaking the viral genome into smaller fragments, sequencing each fragment individually, and then combining the separated fragments into a complete genome sequence using computer algorithms [23]. The Solidarity Trial began in March 2020, immediately after the genome sequence of SARS-CoV-2 was shared.

Because of its antiviral and anti-inflammatory effects, hydroxychloroquine, an antimalarial medication, was being studied and explored as a therapy option for SARS-CoV-2 by March 2020. Hydroxychloroquine was previously intended to treat systemic lupus erythematosus and rheumatoid arthritis [24], and it is speculated to be able to reduce SARS-CoV-2 replication, according to several in vitro investigations. Clinical trials were conducted as a result, although the outcomes were inconsistent. In a French research, 80 patients with an average age of 52.5 years tested positive for COVID-19. They were administered 600 mg of hydroxychloroquine for 10 days, as well as 500 mg of azithromycin on the first day, followed by 250 mg the next four days. Only two patients out of the 80 exhibited no improvement (an 86-year-old patient who died and a 74-year-old patient who was still in ICU at the time of the report). On day five, viral cultures were created from respiratory samples, and 97.5% of them were negative. Furthermore, a day seven PCR demonstrated that 83% of the patients had a reduced viral load when the nasopharyngeal swab was obtained, and 93% had a lower viral load on day eight [25]. Another French trial, on the other hand, had 11 consecutive patients with a mean age of 58.7 years who were treated with the same exact drug combination (600 mg of hydroxychloroquine for 10 days, 500 mg of azithromycin for the first day, then 250 mg for the next four days). The combination appeared to be ineffectual since one patient died, two were brought to the ICU, and the remaining eight were still positive for COVID-19 by nasal swab [26]. In fact, most studies demonstrate that hydroxychloroquine is ineffective, and as a result, the FDA revoked its approval for emergency use on 15 June 2020 due to a lack of efficacy and potential hazards [27] (Figure 1).

Remdesivir, a broad-spectrum antiviral medication used to treat Ebola and other RNA viruses, was the second medicine approved for emergency use. Remdesivir was granted emergency use authorization by the FDA in April 2020 to treat hospitalized COVID-19 cases. Meanwhile, additional research (in vitro and in vivo) was being conducted. Remdesivir, for example, has been proven in vitro to impede the replication of SARS-CoV-1 and MERS-CoV. In vivo investigations on SARS-CoV-1 mice models revealed that early Remdesivir administration has a direct influence on viral load and related lung pathologies [28]. The FDA approved remdesivir as the first medicine in the United States of America to treat hospitalized COVID-19 cases in October of the same year [28] (Figure 1).

Lopinavir/ritonavir, a fixed-dose combination antiretroviral medicine for the treatment of HIV, was one of the medications that showed promise in the treatment of COVID-19 cases. In China, lopinavir/ritonavir and umifenovir were initially indicated for COVID-19 treatment [29]. Observational and randomized controlled trials with Lopinavir/ritonavir, on the other hand, failed to detect a benefit with treatment [30]. Subsequent findings from two randomized controlled trials, RECOVERY [31] and DISCOVERY [32], provided considerable evidence against the use of lopinavir/ritonavir for COVID-19, with no benefits from starting lopinavir/ritonavir treatment early. In another randomized controlled platform study, 199 patients (positive for COVID-19) were randomly assigned to one of two groups. Group A had 99 patients who were given lopinavir/ritonavir (400 mg and 100 mg, respectively) in addition to conventional therapy, and group B consisted of 100 patients who received only conventional care. Group A had a death rate of 19.2% compared to Group B, which had a mortality rate of 25.0% [33]. Indeed, another study reported that lopinavir/ritonavir treatment resulted in worse results when compared to no antiviral treatment [34]. Thus, early lopinavir/ritonavir dosing or lopinavir/ritonavir use in patients with non-severe/non-critical disease revealed little therapeutic benefit and may be hazardous.

In June 2020, dexamethasone was reported to be helpful in lowering the mortality rate of hospitalized COVID-19 patients and was approved to be included in the treatment plan [35] (Figure 1). Many investigations and clinical trials have also validated this conclusion, highlighting that dexamethasone given in addition to standard care (rather than standard care alone) results in a much higher number of days alive and off mechanical ventilation within the first 28 days [36]. Due to a paucity of effective medical interventions and therapies, the FDA approved convalescent plasma EUA as a COVID-19 treatment in August 2020. [37]. It entails using the blood plasma of a recovered patient (in this case, COVID-19) to treat patients with a current infection. It is most effective and promising when taken as a preventative measure or soon after the onset of symptoms [38]. It’s worth noting that it’s currently considered an experimental medicine; therefore, it’s generally utilized in clinical trials. COVID-19 numbers reached a phenomenal 160 million cases in May of the following year (2021). Thus, the FDA granted EUA to the monoclonal antibody therapy: bamlanivimab and etesevimab, casirivimab and imdevimab, and sotrovimab for the treatment of mild to moderate COVID-19 [39]. Further research has shown that monoclonal antibodies such as bamlanivimab, bamlanivimab-etesevimab, casirivimab-imdevimab, and sotrovimab reduced hospitalization rates by 61%, 87%, 72%, and 86%, respectively [40]. As a result, the FDA approved the use of monoclonal antibody combinations as a therapy for COVID-19 patients at high risk. Furthermore, the FDA granted emergency use authorization for two novel oral antiviral agents, nirmatrelvir/ritonavir and molnupiravir, in December 2021 for the treatment of early symptomatic patients with mild to moderate COVID-19 at high risk of progression to severe disease [41]. While early clinical trials showed an improvement in clinical outcomes, the effect was not consistent and was not felt by all demographic and clinical subgroups.

In addition to the previously FDA-authorized medications, the FDA has issued an EUA to different replacement therapies such as multiFiltrate/multiBic/multiPlus and Regiocit replacement solution between April to August 2020, as well as immune modulator drugs such as tocilizumab, baricitinib, and anakinra between June 2021 to November 2022 (Figure 1) [42]. In February 2022, for has issued an EUA to Bebtelovimab (highly potent SARS-CoV-2 spike glycoprotein receptor binding domain (RBD)-specific antibody) for the treatment of mild-to-moderate COVID-19 in adults and pediatric patients (12 years of age and older weighing at least 40 kg) [43]. In April 2023, the FDA issued an EUA for vilobelimab (chimeric monoclonal immunoglobulin G4 antibody) for the treatment of COVID-19 in hospitalized adults requiring mechanical ventilation or artificial life support (Figure 1) [44]. Recently, in November 2023, the FDA also issued EUA for molnupiravir, the orally administered antiviral prodrug that inhibits the replication of RNA viruses via viral error induction, for the treatment of mild-to-moderate coronavirus illness 2019 (COVID-19) (Figure 1) [45].

## 6. Molecular Mechanisms of Action of Anti-COVID-19 Medications

### 6.1. Inhibitors of Viral Entry into the Human Cell

#### 6.1.1. S Protein Inhibitors: Convalescent Plasma and Monoclonal Antibodies

As previously stated, SARS-CoV-2 entrance is dependent on the spike protein binding to the host’s ACE2. Monoclonal antibodies mimic the immunological response of the host to a viral infection. As a result, they bind to the virus’s S protein, rendering it unable to bind to host cells, preventing future infection and lowering the severity of the symptoms. Convalescent plasma also contains antibodies from a recovered COVID-19 patient, which bind to the S protein of SARS-CoV-2 [46] (Table 1). COVID-19 convalescent plasma (CCP) may help COVID-19 through a variety of methods. The administration of neutralizing antibodies (Nabs) against the SARS-CoV-2 virus provides a technique of providing passive and rapid antibody-mediated immunity (AMI) [47]. Opsonization, toxin and viral neutralization, antibody-dependent cellular cytotoxicity (ADCC), complement activation, phagocytosis, and direct antimicrobial actions via oxidant generation have all been associated with AMI [47,48]. Nabs bind to spike1-receptor binding protein (S1-RBD), S1-N-terminal domain, and S2, preventing viral entry and decreasing viral multiplication in SARS-CoV and MERS [49]. Furthermore, other antibody-mediated processes such as complement activation, antibody-dependent cellular cytotoxicity, and/or phagocytosis may contribute to the therapeutic effect of convalescent plasma. Recently, Tian et al. [50] used ELISA and Biolayer Interferometry Binding to demonstrate that one SARS-CoV-specific antibody, CR3022, binds with COVID-19 RBD and, more crucially, that this antibody does not compete with ACE-2 for binding to COVID-19 RBD. COVID-19’s RBD differs significantly from that of SARS-CoV at the C-terminus residues. Although this distinction does not allow COVID-19 to bind the ACE-2 receptor, it does impact NAb cross-reactivity [50]. In addition to NAbs, other protective antibodies found in plasma include immunoglobulin G (IgG) and immunoglobulin M (IgM). Two years after SARS infection, 89% of recovered patients had IgG-specific and NAbs [51]. Furthermore, the maximum concentration of IgM was seen on the ninth day after disease onset, with class change to IgG occurring in the second week [52]. Non-NAbs that bind to the virus but have no effect on its replication capabilities may aid prevention and/or recovery [53].

#### 6.1.2. Inhibitors of Fusional Entry: TMPRSS2 Inhibitors

TMPRSS2 is a protein (enzyme) that is usually present on the surface of respiratory tract cells. Cleaving the spike protein (proteolysis) enables viral penetration. After the cleavage, the virus can fuse into the cell membrane and achieve access. Inhibitors of TMPRSS2 bind to the active site of this enzyme, preventing cleavage of the spike protein and, consequently, virus entry [54] (Table 1). Processing of the TMPRSS2 protein is one of the crucial steps in activating the membrane activity of the SARS-Corona virus-2 S protein [55]. Subsequently, medications that inhibit its proteolytic activity are required to prevent SARS-Corona Virus-2 membrane fusion. Notably, TMPRSS2 is a human protease that, unlike viral protein targets, does not result in drug resistance when used as a therapeutic target [56]. As a result, TMPRSS2 is one of the most promising anti-SARS-CoV-2 treatment targets. TMPRSS2 inhibitor, like many other protease inhibitors, has been reported and demonstrated to prevent virus entry into host cells [57,58]. A recent study used in silico fragment-based drug design to create new compounds for the catalytic site of TMPRSS2 of SARS-CoV-2. Over 500,000 fragments from the enamine database were tested, and ten newly synthesized compounds outperformed reference medications nafamostat and ambroxol in terms of predicted binding scores and free binding energies with the catalytic binding site [59]. A recent group also discovered covalent small-molecule ketobenzothiazole (kbt) TMPRSS2 inhibitors, which are structurally distinct from and have significantly improved activity over the existing known inhibitors camostat and nafamostat by utilizing rational structure-based drug design (SBDD) coupled to substrate specificity screening of TMPRSS2 [60]. The lead compound MM3122 demonstrated great success in blocking host cell entry of a newly developed VSV-SARS-CoV-2 chimeric virus into Calu-3 human lung epithelial cells, inhibiting cytopathic effects induced by SARS-CoV-2 virus in Calu-3 cells, and locking MERS-CoV cell entry [60].

### 6.2. Inhibitors of Endosomal Entry: Hydroxychloroquine

Chloroquine (CQ) and hydroxychloroquine (HCQ) have shown promising effectiveness against SARS-CoV-2. Different mechanisms of action have been proposed for CQ and HCQ involving endocytic pathway interference, sialic acid receptor blockage, restriction of pH-mediated spike (S) protein cleavage at the ACE2 binding site, and cytokine storm prevention (Table 1) [61]. Regarding endocytic pathway interference, CQ accumulates in endosomes and lysosomes and causes pH neutralization, which inhibits the activities of proteases, inhibiting S protein cleavage and, eventually, the process of viral entrance into a host organism [62]. HCQ also blocks SARS-CoV-2 from moving from early endosomes to early lysosomes, which is essential for the release of the viral genome [63]. The increase in pH of lysosomes and endosomes caused by HCQ leads to the production of autophagosomes, which break the S protein and impede membrane fusion [61]. In terms of sialic acid receptor inhibition, both CQ/HCQ were effective in inhibiting sialic acids (particularly the 9-O-SIA variant), which is required to promote SARS-CoV-2 entrance in the upper respiratory route in addition to the previously recognized ACE2 receptor [64]. Regarding the prevention of pH-mediated S protein cleavage at the ACE2 binding site, it was established that CQ/HCQ plays an important role in inhibiting glycosylation of ACE2 receptors, hence reducing SARS-CoV-2 entry into host organisms [65]. Finally, in terms of its action through the prevention of cytokine storm, HCQ inhibits the antigen processing in the antigen-presenting cells (APC) and the presentation of autoantigen mediated by the major histocompatibility complex (MHC) class II to T cells. Due to this, the levels of activated T cells decline, causing a reduction in the production of cytokines generated by T cells and the B cells [61]. Finally, in terms of cytokine storm prevention, HCQ suppresses antigen processing in APC and autoantigen presentation to T cells via the major MHC class II. As a result, the number of activated T cells decreases, resulting in a decrease in the production of cytokines by T and B cells [61].

### 6.3. Inhibitors of Viral Proteases

#### 6.3.1. Inhibitors of Viral Main Protease (M^pro^): Rupintrivir, Lopinavir/Ritonavir and Nirmatrelvir

The mechanism of action of M^pro^ inhibitors is performed in a series of phases. First, the M^pro^ inhibitors attach to the protease’s active site. They then prevent the protease from carrying out its enzymatic activity by interfering with the enzyme’s catalytic mechanism. This, in turn, prevents viral reproduction and spread throughout the body (Table 1) [66]. Rupintrivir is a 3C-protease inhibitor with a lactone molecule in the P1 position that plays an essential role in binding to the active site. Rupintrivir displayed modest inhibition against SARS-CoV-2 M^pro^, with an IC50 value of 68 µM [67]. Lockbaum et al. [68] identify an intriguing rupintrivir binding conformation that shows an alternate mechanism of inhibition. In complexes with other proteases, its fluorophenylalanine group moves to the S1′ subsite, acting as an obstruction between the two catalytic residues. However, due to its relatively high IC50 and observed side effects in clinical trials, several studies classify rupintrivir as a non-potent antiviral [69]. The combination of two HIV-1 protease inhibitors, lopinavir, and ritonavir, was recently reported to be effective against SARS-CoV, and both medications bind well to the SARS-CoV 3C-like protease (SARS-CoV 3CL^pro^) [70]. Using the identified crystal structure of M^pro^ and the Hex program to dock the ligands to the SARS-CoV main proteinase, in silico binding studies revealed that lopinavir and ritonavir could essentially bind to the active site of SARS main proteinase, but their efficacy was predicted to be poor [71]. Nirmatrelvir/ritonavir is another combination of two drugs that have a similar mechanism. The first component, nirmatrelvir, is a peptidomimetic inhibitor of M^pro^, the primary protease of SARS-CoV-2. By inhibiting M^pro^, the virus is prevented from processing the polyprotein precursors required for viral replication [72]. Nirmatrelvir coadministration with ritonavir is essential to raise nirmatrelvir plasma concentrations to therapeutic levels [72] (Table 1).

#### 6.3.2. Inhibitors of Viral Papain-like Protease (PL^pro^)

The M^pro^ and the PL^pro^ are crucial for viral replication and, hence, constitute promising targets for antiviral therapy. PL^pro^ inhibitors work in the same way as M^pro^ inhibitors; however, they target the PL^pro^ enzyme rather than the M^pro^ enzyme [73,74]. 6-Thioguanine (6-TG) is an anti-leukemia and immunosuppressive drug that was recently shown to reduce viral replication of SARS-CoV-2 with an EC50 of 2.13 μM, similar to remdesivir [66]. Although 6-TG decreased PL^pro^ activity directly, it is important to remember that 6-TG’s mode of action as a chemotherapeutic and immunosuppressant is that it is transformed into 6-Thioguanine ribonucleotides and deoxynucleotides and integrated into RNA and DNA. The incorporation of 6-TG-containing ribonucleotides into SARS-CoV-2 RNA could thus represent a secondary antiviral action of 6-TG [75]. Simeprevir, vaniprevir, paritaprevir, and grazoprevir, four clinically authorized hepatitis C protease inhibitors, have recently been found to suppress SARS-CoV-2 PL^pro^ in vitro and viral replication in Vero-E6 cells [74] (Table 1). These HCV medicines can potentially bind into the M^pro^ substrate-binding cleft, according to virtual docking tests [74]. Another study confirmed Grazoprevir and voxilaprevir, two widely used HCV PIs, showed efficacy against SARS-CoV-2 in lung cells. Furthermore, the authors confirmed that the production of HCV PIs with higher potency and plasma concentrations, such as simeprevir, would need to be reinitiated for clinical use, as was the case with remdesivir [76]. In addition to the above-described compounds, three previously discovered naphthalene-based inhibitors, rac3j, rac3k, and rac5c (racemic versions of 3j, 3k, and 5c, respectively), exhibit promising inhibitory efficacy against SARS-CoV-2 PL^pro^. With an in vitro IC50 value of 0.81 μM, Rac5c is the best [77]; at a dosage of 11 μM, rac5c could protect SARS-CoV-2 infected Vero cells from cytopathic impact without inducing cell toxicity in the antiviral assay, suggesting the strong antiviral properties.

### 6.4. Inhibitors of Viral RNA

#### 6.4.1. Inhibitors of RNA Dependent RNA Polymerase (Rdrp): Remdesivir

Remdesivir, which was initially created to treat the Ebola virus, was later revealed to be effective against other RNA viruses, including SARS-CoV-2 because it is a broad-spectrum antiviral medication [28,74]. The drug inhibits the replication of endemic human CoV-229E and CoV-OC43, which cause upper respiratory infection in children but can cause more severe lower respiratory infection in adults with underlying respiratory conditions (i.e., asthma, COPD) and the elderly, as well as PDCoV, a member of the delta coronavirus genus with the most divergent RdRp of any known CoV when compared to SARS- and MERS-CoV [78]. It is a prodrug, which means it is converted into its active form within the body. When it reaches the cell, it converts to remdesivir triphosphate (RTP), which is an ATP analog. RTP subsequently becomes a component of the RNA chain that is developing as a result of RdRp. As illustrated, the RTP stops the developing chain by targeting the RdRp, preventing continued replication [28,74]. An in silico test of the COVID-19 RdRp model revealed that remdesivir was a powerful medication [79]. SARS-CoV and SARS-CoV-2 are both B lineage betacoronaviruses with 96% identical RdRp amino acid sequences, whereas MERS-CoV is a C lineage betacoronavirus with just 71% identity to SARS-CoV-2 [80] (Table 1).

#### 6.4.2. Replication Inhibitor: Molnupiravir

Molnupiravir is a prodrug that is initially degraded by host cell esterase into N4-hydroxy cytidine [81]. The active N4-hydroxycytidine triphosphate (EIDD-1931 triphosphate) is obtained by phosphorylating the resultant alcohol analogue [82]. This phosphorylated counterpart is structurally similar to the native RdRp substrate cytidine or uridine triphosphate. As a result, EIDD-1931 triphosphate suppresses RdRp and, thus, viral replication [83,84]. Because the molnupiravir′s structure is similar to the nitrogenous base of nucleic acids, the amino form may create hydrogen bonds with G (M:G), and the imino form can make hydrogen bonds with A (M:A) [85]. As a result, molnupiravir serves as a template for protein production [81]. The goal is to prevent viral proteins from being produced, which would result in viral cell death [86]. It has been shown to inhibit SARS-CoV-2 replication in human lung tissue, impede SARS-CoV-2 transmission in ferrets, and diminish SARS-CoV-2 RNA in patients. In animal models and people, molnupiravir increases the incidence of viral RNA mutations and affects SARS-CoV-2 replication [87]. This disrupts the replication process because the viral RNA is copied incorrectly, preventing continued infection and propagation of the virus (Table 1).

**Table 1:** General back of the commonly approved anti-COVID-19 drugs.

Drug	Application	Route of Administration	Target	MOA vs. SARS-CoV-2	References
Convalescent plasma	-	Intravenous	S Protein	Binds to the S protein, which prevents viral attachment.	[47,53]
Monoclonal antibodies	-	Intravenous, subcutaneous, intramuscular	S protein	Binds to the S protein, which prevents viral attachment.	[46,48,49,50,51,52]
Camostat	Chronic pancreatitis	Oral	TMPRSS2	A protease inhibitor that prevents SARS-CoV-2 lung cell infection by inhibiting the virus-activating host cell protease TMPRSS2.	[60]
Hydroxychloroquine	Malarial infections	Oral	Multiple	Different mechanisms of action have been proposed involving endocytic pathway interference, sialic acid receptor blockage, restriction of pH-mediated spike (S) protein cleavage at the angiotensin-converting enzyme 2 (ACE2) binding site, and cytokine storm prevention	[61,62,63,64,65]
Rupintrivir	Human rhinoviral (HRV) infections	Nasal	M^pro^	Inhibitors of Viral Main protease (M^pro^).	[67,68,69]
Lopinavir/ritonavir	HIV infections	Oral	M^pro^	Inhibitors of Viral Main protease (M^pro^).	[70,71]
Nirmatrelvir/ritonavir	-	Oral	M^pro^	Inhibitors of Viral Main protease (M^pro^).	[71,72]
6-Thioguanine	Leukaemia	Oral	PL^pro^	Inhibitors of viral papain-like protease (PL^pro^).	[73,75]
Simeprevir, vaniprevir, paritaprevir, and grazoprevir	Chronic HCV infection	Oral	PL^pro^	Inhibitors of viral papain-like protease (PL^pro^).	[76]
Remdesivir	Ebola virus	Intravenous	Rdrp	Inhibitors of RNA Dependent RNA Polymerase (Rdrp)	[78,79,80]
Molnupiravir	Influenza	Oral	viral RNA	Disrupts the replication process because the viral RNA is copied incorrectly	[81,82,83,85,86,87]

## 7. Future Directions and Areas of Research

### 7.1. Flavonoids

Flavonoids are secondary metabolites and compounds generated by plants (fruits, vegetables, cereals, and so on). Flavonoids have been extensively studied for potential health benefits [88,89,90,91,92]. They have anti-inflammatory and antioxidant properties that have been linked to a decreased risk of chronic diseases such as cancer, cardiovascular disease, and neurological disorders [90,92]. Isoquercetin and quercetin are two flavonoids with antioxidant, immune-modulatory, and anti-inflammatory properties [93,94]. Isoquercetin is a monoglycoclated derivative of quercetin that accumulates more in the intestines and thus reaches circulation at higher concentrations than quercetin. Both have broad-spectrum antiviral activities and have reduced cell infection from the Ebola virus, Zika virus, and other viruses [93,94]. An in vitro test revealed that quercetin can inhibit recombinant SARS-CoV PL^pro^ with an IC50 of 8.6 μM [95], leading to further investigation of it as a potential anti-COVID-19 drug. According to a recent study, several flavonoids (including quercetin) have a higher binding affinity (up to −10.60 kcal/mol) than remdesivir (up to 9.50 kcal/mol) [96]. Another study looked at the interaction of flavonoids with ACE2 receptors overexpressed in HEK293 cells and found that the flavonoids are exclusively bound to the ACE2 receptors [97]. Isoquercetin (115 μM) and quercetin (26.3 μM) both inhibited the host endo/lysosomal cysteine protease cathepsins L (CatL), with IC50 values of 26.3 μM and 115 μM, respectively [98]. During phase one clinical studies, no drug-related serious side effects were recorded with the usage of quercetin up to 5 g/d and isoquercetin up to 1 g/d [93]. These flavonoids should be studied further in clinical trials as a potential treatment for SARS-CoV-2.

### 7.2. Inhaled Drugs

Several inhalation treatments have shown promise in laboratory investigations, although they are still in clinical trials. One major cause is that medications taken orally or intravenously do not reach the lungs in sufficient amounts. As a result, inhaled treatments are being investigated as a potentially viable method of COVID-19 treatment [99,100]. This enables for reduced drug dose with fewer side effects [99] while simultaneously increasing concentrations of the desired drug in the lung. Other benefits include a non-invasive delivery method, high membrane permeability, and skipping first-pass metabolism. Inhaled hydroxychloroquine has been demonstrated to be successful in the treatment of COVID-19 while having fewer side effects than oral hydroxychloroquine [100]. Recently, multiple published findings on a small group of patients demonstrated the potential benefits of inhalation treatment, implying that large-scale clinical trials should be conducted. In phase II randomized, double-blind, placebo-controlled studies, for example, inhaled interferon beta-1a produced better results with fewer side effects (44% vs. 22%) [101]. A multicenter, noninterventional cohort analysis of 954 critically ill COVID-19 patients found that inhaled corticosteroids reduced mortality by a statistically meaningful amount [102]. In a multicenter, open-label, multi-arm, randomized, controlled, adaptive platform trial including about 4700 people, it was discovered that inhaled budesonide could shorten recovery time and reduce the chance of death [103]. In a randomized and open-label phase 2 study of 61 COVID-19 patients with mild to severe disease, it was discovered that inhaled ciclesonide eradicated SARS-CoV-2 more effectively than usual therapy [104]. All these trials confirm the potential role of inhalant medications as potent anti-COVID-19 therapies.

### 7.3. Aptamers

Aptamers are nucleic acid-based binding reagents with the same pathogen affinity and specificity as antibodies [105,106]. Researchers recently developed long-lasting and biologically active aptamers that block SARS-CoV-2 infection with high affinity and potency by interrupting the interaction of the S protein RBD-ACE2 receptor. Beyond antibodies, these aptamer-blocking techniques offer a fresh approach to COVID-19 treatment [105,106]. Heterodimerization of modified aptamers targeting nonoverlapping epitopes is a promising technique for increasing potency and minimizing the influence of mutations on medication efficacy. Aptamers have various advantages for treating COVID-19, including cheaper production costs than antibodies, the lack of a cold chain, and the potential to be administered via inhalation [105]. As a result, they are seen to be strong candidates for future anti-COVID-19 medication development. Notable aptamers in the early stages of development are BC 007 and AS1411. Both aptamers passed phase 1 clinical tests [107], and they are now in or have finished phase 2 tests [108]. Both have great safety profiles, as well as good safety and tolerability in very ill patients, who would be the target patients for COVID-19 disease [107,108]. AS1411 has previously gained FDA approval to conduct an efficacy trial for COVID-19 disease [109].

## 8. Conclusions

The COVID-19 pandemic produced by SARS-CoV-2 has put a strain on the global society not seen since World War II. This epidemic put the world’s healthcare infrastructure under strain, creating economic hardship and posing significant challenges in the discovery of treatments. Hundreds of notable articles have been published since the onset of this pandemic to justify the cause of viral spread, viable preventive measures, and future approaches to be used. This review has been created to save our readers’ time and effort by discussing advances in the development of anti-COVID-19 medications. This evaluation includes a wide spectrum of currently used and approved therapies. Furthermore, we went over the molecular mechanisms of action of these medicines in depth. Potential compounds, such as flavonoids and aptamers that could lead to a breakthrough in the creation of new anti-COVID-19 pharmaceuticals have also been identified, and a new route of drug delivery (inhaled medications) appears intriguing but warrants further investigation. Future pandemic threats cannot be avoided, necessitating a more targeted response; biological information must be transferred into specific treatment concepts for testing. Targeting the repurposing of existing drugs is a simple and accessible means of combating such emerging dangers, which is crucial in the early response, but potentially innovative therapies require extensive study and a stable trial setting.

## Data Availability

Not applicable.

## References

[1] Majumder J., Minko T. (2021). Recent developments on therapeutic and diagnostic approaches for COVID-19. AAPS J..

[2] Wu Y.-C., Chen C.-S., Chan Y.-J. (2020). The outbreak of COVID-19: An overview. J. Chin. Med. Assoc..

[3] Gorbalenya A.E., Baker S.C., Baric R.S., de Groot R.J., Drosten C., Gulyaeva A.A., Haagmans B.L., Lauber C., Leontovich A.M., Neuman B.W. (2020). The species Severe acute respiratory syndrome-related coronavirus: Classifying 2019-nCoV and naming it SARS-CoV-2. Nat. Microbiol..

[4] Cucinotta D., Vanelli M. (2020). WHO declares COVID-19 a pandemic. Acta Bio Medica Atenei Parm..

[5] Lu L., Zhong W., Bian Z., Li Z., Zhang K., Liang B., Zhong Y., Hu M., Lin L., Liu J. (2020). A comparison of mortality-related risk factors of COVID-19, SARS, and MERS: A systematic review and meta-analysis. J. Infect..

[6] Richards F., Kodjamanova P., Chen X., Li N., Atanasov P., Bennetts L., Patterson B.J., Yektashenas B., Mesa-Frias M., Tronczynski K. (2022). Economic burden of COVID-19: A systematic review. Clin. Outcomes Res..

[7] Mulabbi E.N., Tweyongyere R., Byarugaba D.K. (2021). The history of the emergence and transmission of human coronaviruses. Onderstepoort J. Vet. Res..

[8] Srivastava N., Saxena S.K., Saxena S.K. (2020). Prevention and Control Strategies for SARS-CoV-2 Infection. Coronavirus Disease 2019 (COVID-19): Epidemiology, Pathogenesis, Diagnosis, and Therapeutics.

[9] Holmes E.C., Goldstein S.A., Rasmussen A.L., Robertson D.L., Crits-Christoph A., Wertheim J.O., Anthony S.J., Barclay W.S., Boni M.F., Doherty P.C. (2021). The origins of SARS-CoV-2: A critical review. Cell.

[10] Umakanthan S., Sahu P., Ranade A.V., Bukelo M.M., Rao J.S., Abrahao-Machado L.F., Dahal S., Kumar H., Kv D. (2020). Origin, transmission, diagnosis and management of coronavirus disease 2019 (COVID-19). Postgrad. Med. J..

[11] World Health Organization (2021). WHO-Convened Global Study of Origins of SARS-CoV-2: China Part.

[12] Wang M.Y., Zhao R., Gao L.J., Gao X.F., Wang D.P., Cao J.M. (2020). SARS-CoV-2: Structure, biology, and structure-based therapeutics development. Front. Cell. Infect. Microbiol..

[13] Singh S.P., Pritam M., Pandey B., Yadav T.P. (2021). Microstructure, pathophysiology, and potential therapeutics of COVID-19: A comprehensive review. J. Med. Virol..

[14] Thomas S. (2020). The structure of the membrane protein of SARS-CoV-2 resembles the sugar transporter semisweet. Pathog. Immun..

[15] Jackson C.B., Farzan M., Chen B., Choe H. (2022). Mechanisms of SARS-CoV-2 entry into cells. Nat. Rev. Mol. Cell Biol..

[16] Moustaqil M., Ollivier E., Chiu H.P., Van Tol S., Rudolffi-Soto P., Stevens C., Bhumkar A., Hunter D.J., Freiberg A.N., Jacques D. (2021). SARS-CoV-2 proteases PL^pro^ and 3CL^pro^ cleave IRF3 and critical modulators of inflammatory pathways (NLRP12 and TAB1): Implications for disease presentation across species. Emerg. Microbes Infect..

[17] Hillen H.S. (2021). Structure and function of SARS-CoV-2 polymerase. Curr. Opin. Virol..

[18] Chams N., Chams S., Badran R., Shams A., Araji A., Raad M., Mukhopadhyay S., Stroberg E., Duval E.J., Barton L.M. (2020). COVID-19: A multidisciplinary review. Front. Public Health.

[19] Saraste J., Prydz K. (2021). Assembly and cellular exit of coronaviruses: Hijacking an unconventional secretory pathway from the pre-Golgi intermediate compartment via the Golgi ribbon to the extracellular space. Cells.

[20] Güner H.R., Hasanoğlu İ., Aktaş F. (2020). COVID-19: Prevention and control measures in community. Turk. J. Med. Sci..

[21] (2021). WHO Solidarity Trial Consortium Repurposed antiviral drugs for COVID-19—Interim WHO solidarity trial results. N. Engl. J. Med..

[22] Safiabadi Tali S.H., LeBlanc J.J., Sadiq Z., Oyewunmi O.D., Camargo C., Nikpour B., Armanfard N., Sagan S.M., Jahanshahi-Anbuhi S. (2021). Tools and techniques for severe acute respiratory syndrome coronavirus 2 (SARS-CoV-2)/COVID-19 detection. Clin. Microbiol. Rev..

[23] Harilal D., Ramaswamy S., Loney T., Suwaidi H.A., Khansaheb H., Alkhaja A., Varghese R., Deesi Z., Nowotny N., Alsheikh-Ali A. (2020). SARS-CoV-2 whole genome amplification and sequencing for effective population-based surveillance and control of viral transmission. Clin. Chem..

[24] Dima A., Jurcut C., Chasset F., Felten R., Arnaud L. (2022). Hydroxychloroquine in systemic lupus erythematosus: Overview of current knowledge. Ther. Adv. Musculoskelet. Dis..

[25] Gautret P., Lagier J.C., Parola P., Meddeb L., Sevestre J., Mailhe M., Doudier B., Aubry C., Amrane S., Seng P. (2020). Clinical and microbiological effect of a combination of hydroxychloroquine and azithromycin in 80 COVID-19 patients with at least a six-day follow up: A pilot observational study. Travel Med. Infect. Dis..

[26] Ibáñez S., Martínez O., Valenzuela F., Silva F., Valenzuela O. (2020). Hydroxychloroquine and chloroquine in COVID-19: Should they be used as standard therapy?. Clin. Rheumatol..

[27] U.S. Food and Drug Administration Coronavirus (COVID-19) Update: FDA Revokes Emergency Use Authorization for Chloroquine and Hydroxychloroquine. FDA News Release. www.fda.gov/news-events/press-announcements/coronavirus-covid-19-update-fda-revokes-emergency-use-authorization-chloroquine.

[28] Lin H.X.J., Cho S., Meyyur Aravamudan V., Sanda H.Y., Palraj R., Molton J.S., Venkatachalam I. (2021). Remdesivir in Coronavirus Disease 2019 (COVID-19) treatment: A review of evidence. Infection.

[29] Sadeghi A., Ali Asgari A., Norouzi A., Kheiri Z., Anushirvani A., Montazeri M., Hosamirudsai H., Afhami S., Akbarpour E., Aliannejad R. (2020). Sofosbuvir and daclatasvir compared with standard of care in the treatment of patients admitted to hospital with moderate or severe coronavirus infection (COVID-19): A randomized controlled trial. J. Antimicrob. Chemother..

[30] Joseph B.A., Dibas M., Evanson K.W., Paranjape G., Vegivinti C.T.R., Selvan P.T., Saravu K., Gupta N., Pulakurthi Y.S., Keesari P.R. (2021). Efficacy and safety of lopinavir/ritonavir in the treatment of COVID-19: A systematic review. Expert. Rev. Anti Infect. Ther..

[31] RECOVERY Collaborative Group (2020). Lopinavir-ritonavir in patients admitted to hospital with COVID-19 (RECOVERY): A randomised, controlled, open-label, platform trial. Lancet.

[32] Ader F., Peiffer-Smadja N., Poissy J., Bouscambert-Duchamp M., Belhadi D., Diallo A., Delmas C., Saillard J., Dechanet A., Mercier N. (2021). An open-label randomized controlled trial of the effect of lopinavir/ritonavir, lopinavir/ritonavir plus IFN-β-1a and hydroxychloroquine in hospitalized patients with COVID-19. Clin. Microbiol. Infect..

[33] Cao B., Wang Y., Wen D., Liu W., Wang J., Fan G., Ruan L., Song B., Cai Y., Wei M. (2020). A trial of lopinavir–ritonavir in adults hospitalized with severe Covid-19. N. Engl. J. Med..

[34] Bosaeed M., Mahmoud E., Alharbi A., Altayib H., Albayat H., Alharbi F., Ghalilah K., Al Arfaj A., AlJishi J., Alarfaj A. (2021). Favipiravir and hydroxychloroquine combination therapy in patients with moderate to severe COVID-19 (FACCT Trial): An open-label, multicenter, randomized. Control. Trial Infect. Dis. Ther..

[35] RECOVERY Collaborative Group (2021). Dexamethasone in hospitalized patients with COVID-19. N. Engl. J. Med..

[36] Tomazini B.M., Maia I.S., Cavalcanti A.B., Berwanger O., Rosa R.G., Veiga V.C., Avezum A., Lopes R.D., Bueno F.R., Silva M.V.A. (2020). Effect of dexamethasone on days alive and ventilator-free in patients with moderate or severe acute respiratory distress syndrome and COVID-19: The CoDEX randomized clinical trial. JAMA.

[37] FDA US (2020). FDA Issues Emergency Use Authorization for Convalescent Plasma as Potential Promising COVID–19 Treatment, Another Achievement in Administration’s Fight Against Pandemic. https://www.fda.gov/news-events/press-announcements/fda-issues-emergency-use-authorization-convalescent-plasma-potential-promising-covid-19-treatment.

[38] Brown B.L., McCullough J. (2020). Treatment for emerging viruses: Convalescent plasma and COVID-19. Transfus. Apher. Sci..

[39] Corti D., Purcell L.A., Snell G., Veesler D. (2021). Tackling COVID-19 with neutralizing monoclonal antibodies. Cell.

[40] Simşek Yavuz S., Komşuoğlu Celikyurt I. (2021). An update of anti-viral treatment of COVID-19. Turk. J. Med. Sci..

[41] Butt A.A., Yan P., Shaikh O.S., Omer S.B., Mayr F.B., Talisa V.B. (2023). Molnupiravir use and 30-day hospitalizations or death in a previously uninfected nonhospitalized high-risk population with COVID-19. J. Infect. Dis..

[42] FDA Coronavirus (COVID-19). Drugs. https://www.fda.gov/drugs/emergency-preparedness-drugs/coronavirus-covid-19-drugs.

[43] FDA Emergency Use Authorization (EUA) for Bebtelovimab (LY-CoV1404) Center for Drug Evaluation and Research (CDER) Review. https://www.fda.gov/media/156396/download.

[44] FDA FDA Authorizes Gohibic (Vilobelimab) Injection for the Treatment of COVID-19. https://www.fda.gov/media/166823/download?attachment.

[45] FDA Lagevrio Letter of Authorization. https://www.fda.gov/media/155053/download?attachment.

[46] Taylor P.C., Adams A.C., Hufford M.M., De La Torre I., Winthrop K., Gottlieb R.L. (2021). Neutralizing monoclonal antibodies for treatment of COVID-19. Nat. Rev. Immunol..

[47] Casadevall A., Pirofski L.A. (2020). The convalescent sera option for containing COVID-19. J. Clin. Investig..

[48] Casadevall A., Dadachova E., Pirofski L.A. (2004). Passive antibody therapy for infectious diseases. Nat. Rev. Microbiol..

[49] Du L., He Y., Zhou Y., Liu S., Zheng B.-J., Jiang S. (2009). The spike protein of SARS-CoV—A target for vaccine and therapeutic development. Nat. Rev. Microbiol..

[50] Tian X., Li C., Huang A., Xia S., Lu S., Shi Z. (2020). Potent binding of 2019 novel coronavirus spike protein by a SARS coronavirus-specific human monoclonal antibody. Emerg. Microbes Infect..

[51] Chaigne B., Mouthon L. (2017). Mechanisms of action of intravenous immunoglobulin. Transfus. Apher. Sci..

[52] Rokni M., Ghasemi V., Tavakoli Z. (2020). Immune responses and pathogenesis of SARS-CoV-2 during an outbreak in Iran: Comparison with SARS and MERS. Rev. Med. Virol..

[53] Bloch E.M., Shoham S., Casadevall A., Sachais B.S., Shaz B., Winters J.L. (2020). Deployment of convalescent plasma for the prevention and treatment of COVID-19. J. Clin. Investig..

[54] Hempel T., Raich L., Olsson S., Azouz N.P., Klingler A.M., Hoffmann M., Pöhlmann S., Rothenberg M.E., Noé F. (2021). Molecular mechanism of inhibiting the SARS-CoV-2 cell entry facilitator TMPRSS2 with camostat and nafamostat. Chem. Sci..

[55] Stopsack K.H., Mucci L.A., Mph S., Antonarakis E.S., Nelson P.S., Kantoff P.W. (2020). TMPRSS2 and COVID-19: Serendipity or opportunity for intervention?. Cancer Discov..

[56] Shen L.W., Mao H.J., Wu Y.L., Tanaka Y., Zhang W. (2017). TMPRSS2: A potential target for treatment of influenza virus and coronavirus infections. Biochimie.

[57] Hussain M., Jabeen N., Amanullah A., Baig A.A., Aziz B., Shabbir S., Raza F., Uddin N. (2020). Molecular docking between human tmprss2 and SARS-CoV-2 spike protein: Conformation and intermolecular interactions. AIMS Microbiol..

[58] Touret F., Gilles M., Barral K., Nougairède A., Van Helden J., Decroly E., De Lamballerie X., Coutard B. (2020). In vitro screening of a FDA approved chemical library reveals potential inhibitors of SARS-CoV-2 replication. Sci. Rep..

[59] Alzain A.A., Elbadwi F.A., Alsamani F.O. (2022). Discovery of novel TMPRSS2 inhibitors for COVID-19 using in silico fragment-based drug design, molecular docking, molecular dynamics, and quantum mechanics studies. Inform. Med. Unlocked.

[60] Mahoney M., Damalanka V.C., Tartell M.A., Chung D.H., Lourenço A.L., Pwee D., Mayer Bridwell A.E., Hoffmann M., Voss J., Karmakar P. (2021). A novel class of TMPRSS2 inhibitors potently block SARS-CoV-2 and MERS-CoV viral entry and protect human epithelial lung cells. Proc. Natl. Acad. Sci. USA.

[61] Satarker S., Ahuja T., Banerjee M., E V.B., Dogra S., Agarwal T., Nampoothiri M. (2020). Hydroxychloroquine in COVID-19: Potential mechanism of action against SARS-CoV-2. Curr. Pharmacol. Rep..

[62] Yang N., Shen H.M. (2020). Targeting the endocytic pathway and autophagy process as a novel therapeutic strategy in COVID-19. Int. J. Biol. Sci..

[63] Liu J., Cao R., Xu M., Wang X., Zhang H., Hu H., Li Y., Hu Z., Zhong W., Wang M. (2020). Hydroxychloroquine, a less toxic derivative of chloroquine, is effective in inhibiting SARS-CoV-2 infection in vitro. Cell Discov..

[64] Fantini J., Di Scala C., Chahinian H., Yahi N. (2020). Structural and molecular modelling studies reveal a new mechanism of action of chloroquine and hydroxychloroquine against SARS-CoV-2 infection. Int. J Antimicrob. Agents.

[65] Pahan P., Pahan K. (2020). Smooth or risky revisit of an old malaria drug for COVID-19?. J. Neuroimmune Pharmacol..

[66] Pang X., Xu W., Liu Y., Li H., Chen L. (2023). The research progress of SARS-CoV-2 main protease inhibitors from 2020 to 2022. Eur. J. Med. Chem..

[67] Vatansever E.C., Yang K., Kratch K.C., Drelich A., Cho C.-C., Mellott D.M., Xu S., Tseng C.-T.K., Liu W.R. (2021). Bepridil is potent against SARS-CoV-2 in vitro. Proc. Natl. Acad. Sci. USA.

[68] Lockbaum G.J., Henes M., Lee J.M., Timm J., Nalivaika E.A., Thompson P.R., Yilmaz N.K., Schiffer C.A. (2021). Pan-3C protease inhibitor rupintrivir binds SARS-CoV-2 main protease in a unique binding mode. Biochemistry.

[69] Ma C., Sacco M.D., Hurst B., Townsend J.A., Hu Y., Szeto T., Zhang X., Tarbet B., Marty M.T., Chen Y. (2020). Boceprevir, GC-376, and calpain inhibitors II, XII inhibit SARS-CoV-2 viral replication by targeting the viral main protease. Cell Res..

[70] Nutho B., Mahalapbutr P., Hengphasatporn K., Pattaranggoon N.C., Simanon N., Shigeta Y., Hannongbua S., Rungrotmongkol T. (2020). Why are lopinavir and ritonavir effective against the newly emerged coronavirus 2019? Atomistic insights into the inhibitory mechanisms. Biochemistry.

[71] Zhang X.W., Yap Y.L. (2004). Old drugs as lead compounds for a new disease? Binding analysis of SARS coronavirus main proteinase with HIV, psychotic and parasite drugs. Bioorg. Med. Chem..

[72] Lam C., Patel P. (2023). Nirmatrelvir-Ritonavir.

[73] Swaim C.D., Dwivedi V., Perng Y.-C., Zhao X., Canadeo L.A., Harastani H.H., Darling T.L., Boon A.C.M., Lenschow D.J., Kulkarni V. (2021). 6-Thioguanine blocks SARS-CoV-2 replication by inhibition of PL^pro^. iScience.

[74] Bafna K., White K., Harish B., Rosales R., Ramelot T.A., Acton T.B., Moreno E., Kehrer T., Miorin L., Royer C.A. (2021). Hepatitis C virus drugs that inhibit SARS-CoV-2 papain-like protease synergize with remdesivir to suppress viral replication in cell culture. Cell Rep..

[75] Swaim C.D., Perng Y.C., Zhao X., Canadeo L.A., Harastani H.H., Darling T.L., Boon A.C., Lenschow D.J., Huibregtse J.M. (2020). 6-Thioguanine blocks SARS-CoV-2 replication by inhibition of PLpro protease activities. BioRxiv.

[76] Gammeltoft K.A., Zhou Y., Duarte Hernandez C.R., Galli A., Offersgaard A., Costa R., Pham L.V., Fahnøe U., Feng S., Scheel T.K. (2021). Hepatitis C virus protease inhibitors show differential efficacy and interactions with remdesivir for treatment of SARS-CoV-2 in vitro. Antimicrob. Agents Chemother..

[77] Klemm T., Ebert G., Calleja D.J., Allison C.C., Richardson L.W., Bernardini J.P., Lu B.G.C., Kuchel N.W., Grohmann C., Shibata Y. (2020). Mechanism and inhibition of the papain-like protease, PLpro, of SARS-CoV-2. EMBO J..

[78] Brown A.J., Won J.J., Graham R.L., Dinnon K.H., Sims A.C., Feng J.Y., Cihlar T., Denison M.R., Baric R.S., Sheahan T.P. (2019). Broad spectrum antiviral remdesivir inhibits human endemic and zoonotic deltacoronaviruses with a highly divergent RNA dependent RNA polymerase. Antivir. Res..

[79] Elfiky A.A. (2020). Anti-HCV, nucleotide inhibitors, repurposing against COVID-19. Life Sci..

[80] Gordon C.J., Tchesnokov E.P., Woolner E., Perry J.K., Feng J.Y., Porter D.P., Götte M. (2020). Remdesivir is a direct-acting antiviral that inhibits RNA-dependent RNA polymerase from severe acute respiratory syndrome coronavirus 2 with high potency. J. Biol. Chem..

[81] Teli D., Balar P., Patel K., Sharma A., Chavda V., Vora L. (2023). Molnupiravir: A versatile prodrug against SARS-CoV-2 variants. Metabolites.

[82] Wang Z., Yang L. (2022). Broad-spectrum prodrugs with anti-SARS-CoV-2 activities: Strategies, benefits, and challenges. J. Med. Virol..

[83] Zarenezhad E., Marzi M. (2022). Review on molnupiravir as a promising oral drug for the treatment of COVID-19. Med. Chem. Res..

[84] Parra-Lucares A., Segura P., Rojas V., Pumarino C., Saint-Pierre G., Toro L. (2022). Emergence of SARS-CoV-2 variants in the world: How could this happen?. Life.

[85] Menéndez-Arias L. (2021). Decoding molnupiravir-induced mutagenesis in SARS-CoV-2. J. Biol. Chem..

[86] Gordon C.J., Tchesnokov E.P., Schinazi R.F., Götte M. (2021). Molnupiravir promotes SARS-CoV-2 mutagenesis via the RNA template. J. Biol. Chem..

[87] Kabinger F., Stiller C., Schmitzová J., Dienemann C., Kokic G., Hillen H.S., Höbartner C., Cramer P. (2021). Mechanism of molnupiravir-induced SARS-CoV-2 mutagenesis. Nat. Struct. Mol. Biol..

[88] Khalifa H.O., Kamimoto M., Shimamoto T., Shimamoto T. (2015). Antimicrobial effects of blueberry, raspberry, and strawberry aqueous extracts and their effects on virulence gene expression in *Vibrio cholerae*. Phytother. Res..

[89] Sorour S.S., Abou Asa S., Elhawary N.M., Ghazy E.W., Abd El Latif A., El-Abasy M.A., Khalifa H.O. (2018). Anticoccidial and hepatoprotective effects of artemisinin liquid extract, cinnamon essential oil and clove essential oil against *Eimeria stiedae* infection in rabbits. Trop. Biomed..

[90] Abd El-Hafeez A.A., Khalifa H.O., Elgawish R.A., Shouman S.A., Abd El-Twab M.H., Kawamoto S. (2018). *Melilotus indicus* extract induces apoptosis in hepatocellular carcinoma cells via a mechanism involving mitochondria-mediated pathways. Cytotechnology.

[91] Abd El-Hafeez A.A., Marzouk H.M.M., Abdelhamid M.A., Khalifa H.O., Hasanin T.H., Habib A.G., Abdelwahed F.M., Barakat F.M., Bastawy E.M., Abdelghani E.M. (2022). Anti-cancer effect of *Hyoscyamus muticu*s extract via its activation of Fas/FasL-ASK1-p38 pathway. Biotechnol. Bioprocess Eng..

[92] Khalifa H.O.A. (2016). Molecular Pharmacological Studies on Multidrug-Resistant Bacteria: Analysis of Antimicrobial Resistance Mechanisms and Evaluation of Antimicrobial and Antivirulence Activities of Novel Plant Extracts. Ph.D. Thesis.

[93] Mbikay M., Chrétien M. (2022). Isoquercetin as an anti-COVID-19 medication: A potential to realize. Front. Pharmacol..

[94] Aghababaei F., Hadidi M. (2023). Recent advances in potential health benefits of quercetin. Pharmaceuticals.

[95] Park J.Y., Yuk H.J., Ryu H.W., Lim S.H., Kim K.S., Park K.H., Ryu Y.B., Lee W.S. (2017). Evaluation of polyphenols from *Broussonetia papyrifera* as coronavirus protease inhibitors. J. Enzyme Inhib. Med. Chem..

[96] Hiremath S., Kumar H.V., Nandan N., Mantesh M. (2021). In silico docking analysis revealed the potential of phytochemicals present in *Phyllanthus amarus* and *Andrographis paniculata*, used in Ayurveda medicine in inhibiting SARS-CoV-2. 3 Biotech.

[97] Zhan Y., Ta W., Tang W., Hua R., Wang J., Wang C., Lu W. (2021). Potential antiviral activity of isorhamnetin against SARS-CoV-2 spike pseudotyped virus in vitro. Drug Dev. Res..

[98] Ramalho S.D., de Sousa L.R., Burger M.C., Lima M.I.S., da Silva M.F.D.G., Fernandes J.B., Vieira P.C. (2015). Evaluation of flavonols and derivatives as human cathepsin B inhibitor. Nat. Prod. Res..

[99] Pasqua E., Hamblin N., Edwards C., Baker-Glenn C., Hurley C. (2022). Developing inhaled drugs for respiratory diseases: A medicinal chemistry perspective. Drug Discov. Today.

[100] de Reus Y.A., Hagedoorn P., Sturkenboom M.G.G., Grasmeijer F., Bolhuis M.S., Sibum I., Kerstjens H.A.M., Frijlink H.W., Akkerman O.W. (2022). Tolerability and pharmacokinetic evaluation of inhaled dry powder hydroxychloroquine in healthy volunteers. PLoS ONE.

[101] Monk P.D., Marsden R.J., Tear V.J., Brookes J., Batten T.N., Mankowski M., Gabbay F.J., Davies D.E., Gabbay F.J., Davies D.E. (2021). Safety and efficacy of inhaled nebulised interferon beta-1a (SNG001) for treatment of SARS-CoV-2 infection: A randomised, double-blind, placebo- controlled, phase 2 trial. Lancet Respir. Med..

[102] Al Sulaiman K., Aljuhani O., Al Aamer K., Al Shaya O., Al Shaya A., Alsaeedi A.S., Alhubaishi A., Altebainawi A.F., Al Harthi A., Albelwi S. (2022). The role of inhaled corticosteroids (ICS) in critically ill patients with COVID-19: A multicentre, cohort study. J. Intensive Care Med..

[103] Yu L.-M., Bafadhel M., Dorward J., Hayward G., Saville B.R., Gbinigie O., Van Hecke O., Ogburn E., Evans P.H., Thomas N.P.B. (2021). Inhaled budesonide for COVID-19 in people at high risk of complications in the community in the UK (PRINCIPLE): A randomised, controlled, open- label, adaptive platform trial. Lancet.

[104] Song J.Y., Yoon J.G., Seo Y.B., Lee J., Eom J.S., Lee J.S., Choi W.S., Lee E.Y., Choi Y.A., Hyun H.J. (2021). Ciclesonide inhaler treatment for mild-to-moderate COVID-19: A randomized, open-label, Phase 2 trial. J. Clin. Med..

[105] Robinson P.C., Liew D.F., Tanner H.L., Grainger J.R., Dwek R.A., Reisler R.B., Steinman L., Feldmann M., Ho L.P., Hussell T. (2022). COVID-19 therapeutics: Challenges and directions for the future. Proc. Natl. Acad. Sci. USA.

[106] Mahmoudi A., Alavizadeh S.H., Hosseini S.A., Meidany P., Doagooyan M., Abolhasani Y., Saadat Z., Amani F., Kesharwani P., Gheybi F. (2023). Harnessing aptamers against COVID-19: A therapeutic strategy. Drug Discov. Today.

[107] Becker N.-P., Haberland A., Wenzel K., Göttel P., Wallukat G., Davideit H., Schulze-Rothe S., Hönicke A.-S., Schimke I., Bartel S. (2020). Three-Part, randomised study to investigate the safety, Tolerability, Pharmacokinetics and Mode of action of BC 007, neutraliser of pathogenic auto-antibodies against G-Protein coupled receptors in healthy, young and elderly subjects. Clin. Drug Investig..

[108] Rosenberg J.E., Bambury R.M., Van Allen E.M., Drabkin H.A., Lara P.N., Harzstark A.L., Wagle N., Figlin R.A., Smith G.W., Garraway L.A. (2014). A phase II trial of AS1411 (a novel nucleolin-targeted DNA aptamer) in metastatic renal cell carcinoma. Investig. New Drugs.

[109] Haberland A., Müller J. (2022). Aptamers against COVID-19: An untested opportunity. Mini Rev. Med. Chem..

